# Development, Calibration, and Validation of a U.S. White Male Population-Based Simulation Model of Esophageal Adenocarcinoma

**DOI:** 10.1371/journal.pone.0009483

**Published:** 2010-03-01

**Authors:** Chin Hur, Tristan J. Hayeck, Jennifer M. Yeh, Ethan B. Richards, Stuart J. Spechler, G. Scott Gazelle, Chung Yin Kong

**Affiliations:** 1 Massachusetts General Hospital Institute for Technology Assessment, Boston, Massachusetts, United States of America; 2 Massachusetts General Hospital Gastrointestinal Unit, Boston, Massachusetts, United States of America; 3 Harvard Medical School, Boston, Massachusetts, United States of America; 4 Harvard School of Public Health, Boston, Massachusetts, United States of America; 5 University of Texas Southwestern Medical Center, Dallas, Texas, United States of America; Albert Einstein College of Medicine, United States of America

## Abstract

**Background:**

The incidence of esophageal adenocarcinoma (EAC) has risen rapidly in the U.S. and western world. The aim of the study was to begin the investigation of this rapid rise by developing, calibrating, and validating a mathematical disease simulation model of EAC using available epidemiologic data.

**Methods:**

The model represents the natural history of EAC, including the essential biologic health states from normal mucosa to detected cancer. Progression rates between health states were estimated via calibration, which identified distinct parameter sets producing model outputs that fit epidemiologic data; specifically, the prevalence of pre-cancerous lesions and EAC cancer incidence from the published literature and Surveillance, Epidemiology, and End Results (SEER) data. As an illustrative example of a clinical and policy application, the calibrated and validated model retrospectively analyzed the potential benefit of an aspirin chemoprevention program.

**Results:**

Model outcomes approximated calibration targets; results of the model's fit and validation are presented. Approximately 7,000 cases of EAC could have been prevented over a 30-year period if all white males started aspirin chemoprevention at age 40 in 1965.

**Conclusions:**

The model serves as the foundation for future analyses to determine a cost-effective screening and management strategy to prevent EAC morbidity and mortality.

## Introduction

The vast majority of esophageal cancers are either squamous cell carcinoma (SCC) or adenocarcinoma (EAC). Although esophageal SCC incidence has been declining in the U.S. and other parts of the western world, EAC incidence has experienced an alarming, greater than five-fold, increase over the past three decades [1]. This makes EAC the solid tumor with the most rapidly increasing incidence. Although the absolute number of EAC cases per year remains too low to screen the general population [2], targeted screening of high risk individuals may be appropriate. Heartburn, the primary symptom of gastroesophageal reflux disease (GERD), affects 60 million Americans [3] and can lead to Barrett's esophagus (BE). BE is a pre-malignant condition associated with the greatest risk (30–125 times) of developing EAC [4]. The management of these patients has become a significant public health issue because of the significant number of individuals affected by GERD and BE. The development of clinical and policy guidelines for disease management requires evidence, such as results from clinical trials. However, the relatively low rate of progression to cancer [5] has made clinical trials with cancer endpoints challenging because of the large number of subjects and long follow-up period required. Additionally, no single clinical study can evaluate all the possible screening and management strategies, both current and pending, that attempt to diminish the morbidity caused by EAC. These factors have presented obstacles in developing an acceptable screening and surveillance strategy.

A lack of quality clinical data from controlled studies of necessary duration has also limited our understanding of the natural history of EAC. Mathematical simulation models constructed by integrating the best available biologic, epidemiologic, and clinical data can be useful in this circumstance [6]. Such models improve our overall understanding of the natural history of EAC, including the ability to estimate the unobservable transitions between health states and can highlight areas to target for future research. The construction of an EAC simulation model will furthermore provide the necessary foundation for cost-effectiveness analyses which can inform clinical and policy decisions.

The aim of this article is to detail the construction of an EAC model including: descriptions of model structure and inputs; calibration endpoints and methodology; and model validation. Additionally, for illustrative purposes, we provide a policy application of the EAC model by retrospectively analyzing the potential effects of a hypothetical national aspirin chemoprevention program on EAC incidence. Epidemiologic analyses have found that aspirin use is associated with a 50% decreased rate of EAC [7]. We applied our natural history model as a systematic method to estimate the lives saved under a hypothetical national aspirin chemoprevention program. In the future, this validated model can serve as the foundation for analyses that determine a cost-effectiveness screening and management strategy for the prevention of EAC morbidity and mortality.

## Materials and Methods

### Overview

The data sources for model development were from the published literature and the National Cancer Institute's Surveillance, Epidemiology, and End Results (SEER) databases [2]. We describe phases of the model development process: defining the model structure or health states; specification of model parameters and assumptions; and estimation of parameters by calibrating the model to published studies and SEER data. Calibration is the process of inferring the unknown transition probabilities in the underlying biological processes by fitting the model output to the empirical data (i.e. calibration targets). Model calibration can thus identify a series of good-fitting parameters sets that are consistent with the empirical data.

### Model Structure

**Figure 1:**
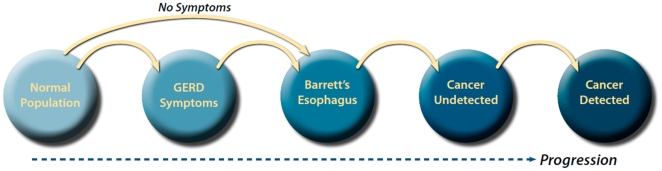
Schematic of the Model Structure.

We developed a Markov state transition simulation model of esophageal carcinogenesis with six health states including Normal, GERD Symptoms, BE, Undetected Cancer, Detected Cancer and Death as shown in Figure 1. Due to the complications of age-dependent all-cause mortality and non-constant transition rates between some health states, no exact analytical solution was found for this model; hence we applied a simulation approach to solve for the transition rates between the various pre-cancerous and cancerous states. The model was programmed in Visual C++ using the Microsoft.NET Version 2.0 Framework (Redmond, WA).

At the start of a simulation, a cohort of 20 year old white men enters the model from the Normal health state. Individuals in a health state can progress to the next state based on an annual transition probability. At any point in the simulation, individuals can die (and enter the absorbing Death state) from age-dependent all-cause mortality in the pre-cancerous states. However, once individuals develop cancer, they are subjected to cancer-specific mortality rates. Since a significant percentage of patients with BE report no GERD symptoms [8], the model also allows for progression from the Normal health state directly to the BE health state.

### Model Assumptions

In accordance with the consensus reached during an NIH-sponsored conference [R13 DK079674] regarding future disease models of BE and EAC [9], we assumed there was no regression among health states; although, regression from some of the health states may be biologically plausible. Additionally, other health states such as dysplasia (both low grade and high grade) were not included. These assumptions allowed us to maintain a parsimonious model, which simplified the model calibration process.

Although not depicted in our basic schematic, the BE state was further sub-classified into Long Segment BE (LSBE) and Short Segment BE (SSBE) by a ratio of 1∶3 in agreement with the range found in the literature [10], [11], [12]. We used a surface area rationale that assumed the progression rates for SSBE was half of that for LSBE, or at a 1∶2 ratio because of the limited data in the existing literature regarding cancer progression in SSBE [13]. We only modeled white men as they account for the majority of the cases of EAC in the U.S. [1]. See more details regarding this limitation and others in the Discussion section. For the aspirin chemoprevention analysis, the model only included the effects of progression rate reduction by aspirin from BE to Undetected Cancer.

### Parameterization

To aid in establishing the parameter boundaries in the model, a comprehensive literature search was performed. Due to limited publications in this area, only a few transition probabilities were found in the published data; see Table 1.

**Table 1:** Model Inputs: Selected Parameters Estimates.

Parameter	Value	Range	References
*Population Prevalence (%)*			
GERD Symptoms Prevalence	18.6	17.6–19.9	[3], [12], [15], [17], [18], [19], [20], [21], [22], [23], [24]
BE Prevalence	4.2	0.8–25	[10], [11], [12], [25], [26], [27], [28], [29], [31], [43]
*Transition Probabilities*			
Normal to GERD Symptoms	Derived From Calibration	Derived From Calibration	
Normal to BE	″	″	
GERD to BE	″	″	
BE to Undetected CA	″	″	
Undetected CA to Detected CA	4.5 years	4–9 years	[32], [38]
*Chemoprevention*			
Aspirin Effect on EAC	50% Reduction		[36]

This table describes the literature-derived initial ranges and values used as model input parameters. Population prevalences of GERD and BE are not themselves model parameters, but they are used to derive values for transition or progression from the “Normal” state to the “GERD” and “BE” states, respectively.

Derivation of these prevalence values are described in the Appendix S1.

**Abbreviations**: GERD = gastroesophageal reflux disease; BE = Barrett's esophagus.

Due to the lack of published data that provide GERD symptom prevalence stratified by age, we started with an overall prevalence of approximately 20% [14]; we then performed a weighted regression on data from all available studies [3], [12], [15], [16], [17], [18], [19], [20], [21], [22], [23], [24] that presented the mean age and GERD symptoms prevalence of the study group. The regression resulted in an overall prevalence of 18.6% (weighted average), which is consistent with the US estimate by Locket et al [14] which reported the GERD symptom prevalence in a range between 17.7%–21.9% (95% confidence intervals). Further information on this can be found in the Appendix S1.

The prevalence of BE in the population published in the literature has a wide range (0.8–25%) [10], [11], [12], [25], [26], [27], [28], [29], [30], [31]. The evolving definition of BE with the inclusion of short-segment and ultra-short segment BE has been one of the causes for this ambiguity. The BE prevalences used as the calibration endpoints for our model focused on three specific studies.

The two largest and methodologically most rigorous studies that estimated population prevalences of BE were published by Rex et al [11] and Ronkainen et al [12]. It has been hypothesized that differing diagnostic criteria (e.g. endoscopic biopsy methods) could have contributed to the divergent findings. Consequently, we chose to average the results of these two similar sized studies and estimated that the overall prevalence of BE in the US population was approximately 4.2% in the base case. We then performed a linear regression on the age-specific BE prevalences from the Cameron article and recorded the resulting regression slope. Further information on this can be found in the Appendix S1.

Since the “Undetected Cancer” state is by definition unobservable, the transition rates to and from this state are associated with a high level of uncertainty. However, some data on the *sojourn time* of “Undetected Cancer” were found in the literature. For our analysis, *sojourn time* was defined as the length of time it would take for an undetected cancer to progress and to become detected due to clinical symptoms such as dysphagia (difficulties swallowing) or weight loss. A study estimated that the time from endoscopically detectable EAC to clinical symptoms was on average 4–5 years [32]. We reasoned that the Undetected Cancer state would likely occur before the endoscopically detectable EAC used in the study. Accordingly, to account for the earlier occurrence of Undetected Cancer, the range was broadened to 4–9 years.

### Calibration Targets

Calibration targets were derived using published data and included age-specific prevalences of individuals in the U.S. with GERD Symptoms and BE as well as age-specific U.S. incidences of EAC based on SEER data [2]. See Table 2 for summary. Also see the *Appendix S1* for additional details regarding the methods used for model calibration and validation.

**Table 2:** Calibration Targets.

Age Group	EAC Incidence	BE Prevalence*	GERD Symptom Prevalence**
20–29 yrs	0.0	1.7	17.6
30–39 yrs	0.3	2.5	18.0
40–49 yrs	0.6	3.3	18.4
50–59 yrs	3.3	4.1	18.8
60–69 yrs	7.4	5.0	19.1
70–79 yrs	9.3	5.8	19.5

All data is from 1986, a representative year, chosen because it is the midpoint of our time period.

*Overall BE prevalence for the population is 4.2%.

**Overall GERD Symptom Prevalence is 18.6%.

**Abbreviations**: EAC = Esophageal Adenocarcinoma; BE = Barrett's esophagus; GERD = gastroesophageal reflux disease.

We used SEER incidence data from calendar year 1973–2000 for model calibration; 2001–2005 data were reserved for validation. The time span of 28 years used in calibration allowed the model to capture the rapidly rising EAC incidence. In contrast, we assumed that the other primary calibration targets of GERD Symptom and BE prevalence were not subject to any secular trends (other than those created by demographic shifts such as changes in population age distributions over time) and did not change in the 28 year calibration period. The calibration targets are further stratified into 10-year age groups between ages of 20 and 79.

Table 2 provides an illustrative representative year, as SEER incidence changes with calendar year, and presents EAC incidence and BE and GERD symptom prevalences by age group for the calendar year of 1986.

### Empirical Calibration

We used calibration to identify parameters sets of transition probabilities to ensure that the model outputs were consistent with the three targets of cancer incidence (age-specific SEER data from 1973–2000) and prevalences of two biologic predecessor health states (age-specific BE and GERD symptom prevalence). The parameter search process, described below, produced numerous sets of distinct transition probabilities resulting in model outputs that had a good “fit” to the calibration targets. The ranges of the resulting transition probabilities represent parameter uncertainty.

#### GOF metric and assessment of fit

To assess the adequacy of the model parameters derived from calibration, the Chi-Squared **G**oodness **o**f **F**it (GOF) was used as the metric to compare model output to the calibration targets for each parameter set used (see *Appendix S1* for more details). The best-fitting parameter sets were defined as those with the lowest GOF scores. The *best* 1% (out of 10^5^) or 1,000 parameter sets were selected and used for model validation and the aspirin analysis. The best 1% of the parameter sets was chosen instead of the top 5% to handle noise in fitting the three endpoints while still leaving enough parameter sets to perform analysis. Additionally, analysis of these *best* 1,000 parameters sets allowed us to infer a likely range of minimum and maximum values for each transition probability.

#### Systematic search algorithm

In order to systematically search the parameter space for model calibration in a reproducible manner, we used the *Simulated Annealing* (SA) algorithm, a commonly used engineering optimization technique [33]. In each calibration run, 10^5^ search simulations were performed. At the beginning of the parameter search, random parameter values were selected within the allowable parameter range. The SA algorithm searches for the global minimal GOF score and has been shown to efficiently sample the parameter space of simulation models with large number of parameters [33].

#### Secular trend function

A prominent increase in the U.S. EAC incidence has been observed over the past few decades. In order to account for this rapid change in incidence, our model required an additional secular function for the model output to reproduce SEER cancer incidence. The options for where to incorporate a secular trend function into the model were limited because we assumed a stable prevalence of BE from 1973–2000. Additionally, because the stage distributions of detected cancers from SEER data have remained relatively constant since 1973, we assumed that the transition rate from Undetected Cancer to Detected Cancer was also stable over this time period. Once the BE prevalence (pre-cursor) and the transition from Undetected to Detected CA were “fixed”, only the transition probability from BE to Undetected Cancer could accommodate an additional secular factor for the model output to accurately reproduce SEER cancer incidence. Other cancer models and analyses have described the incorporation of similar functions in order to approximate clinical data that exhibit a secular trend [34], [35]. Using different secular trend functions, the model was fit to the calibration targets over the 28 year span using a three phase calibration procedure described below. A linear function was ultimately chosen because it provided the best fit to the SEER incidence and was also the most simple. See *Appendix S1* for additional details regarding both the secular trend function and three phase calibration.

#### Three phase calibration

The calibration process utilized three target endpoints: GERD symptom prevalence, BE prevalence, and SEER cancer incidence. The calibration procedure needed to address the substantial heterogeneity in regards to both the sample size (e.g. SEER data based a significant percentage of the U.S. population) and data quality of the three calibration targets. As a result, we chose a three phase calibration process to sequentially fit each calibration target. Since the sequence of calibration could affect the process and outcome, the biologic progression or Forward order (GERD Symptoms→BE→EAC) and the Reverse order (EAC→BE→GERD symptoms) were tested to assess the impact of order and to determine which was superior.

For each phase of the calibration, 10^5^ SA parameter search simulations were performed (one calibration run). The better parameters sets were those with the 10% lowest GOF scores. Next, the transition probabilities in the model were constrained to a range limited by the minimum and maximum values of the better parameter sets. For the Forward order, this process was first performed to calibrate to GERD Symptom prevalence, followed by BE prevalence, and then with final calibration to EAC incidence.

#### Validation

SEER data from 2001–2005 were reserved for model validation. After the model was calibrated to the three targets from 1973–2000, the *best* 1,000 parameter sets selected from the calibration process were used in the model to project the EAC incidence for the U.S. population from 2001 to 2005. Since the number of years in these two periods (28 years in the calibration years and 5 years in the validation set) were unequal, the scores were divided by the number of years to produce an adjusted GOF score.

Our criterion to confirm validation was if the average adjusted GOF scores of the best 1,000 parameter sets for the validation years was less than 150% (or not inferior by more than 50%) of the value for the calibration years.

### Aspirin Simulation: Illustrative Analysis of Model Output

#### Overview

The use of non-steroidal anti-inflammatory drugs (NSAIDs), particularly aspirin, could potentially have a significant impact on the incidences of EAC. We performed a simulation experiment in a hypothetical scenario to explore the impact of using aspirin as a chemoprevention for EAC, providing an illustration of the model utilization in clinical and public health impact. The model analysis assumed all men at age 40 in the U.S. initiated aspirin in the 1960s (run-in period prior to 1973 when the analysis formally begins) and continued treatment until death for chemoprevention against EAC. The protective effects of aspirin were incorporated into the model to compare the EAC cancer incidence with aspirin against the original model outputs without aspirin. Model simulations used the *best* 1,000 parameters sets as determined in the prior calibration process.

Based on epidemiologic studies, aspirin has been associated with approximately a 50% decrease in EAC prevalence [36]. To incorporate these data into an effect on transition rates from BE to undetected cancer, we assumed a constant effect and used a hazard function to translate a 50% decreased cancer rate at the end of a 5 year period into a 13% decreased rate in annual transitions. 1,000 simulations were run using the *best* parameter sets from years 1973–2005 and the predicted EAC incidence for the white male population were estimated for each simulation; these incidences were then averaged to produce a composite estimate. Sensitivity analysis was performed by varying the efficacy of aspirin from the base case rate of a 50% reduction, with a range from 30–70% tested.

## Results

### Model Fit to Calibration Targets

**Figure 2:**
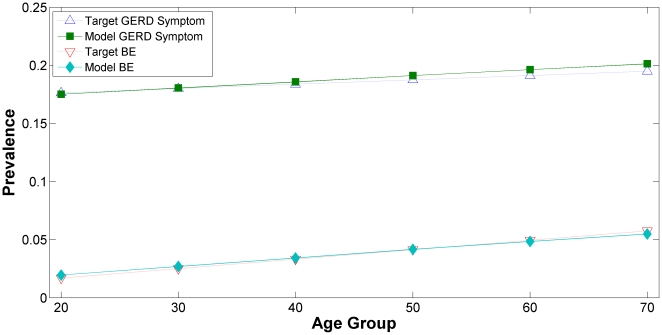
Calibration Targets: GERD Symptoms and BE Prevalences by Age Groups.

The model fits to our first two calibration targets, GERD Symptom and BE prevalences are presented in Figure 2. GERD Symptom prevalence was plotted as a function of specific age group in the upper portion of the graph and BE prevalence was shown in the lower portion. The fits for the GERD Symptom prevalence and BE prevalence are good as model outputs are in agreement with the observed data.

**Figure 3:**
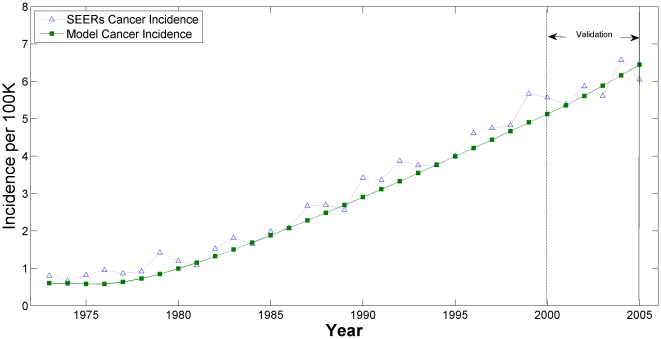
Annual Esophageal Adenocarcinoma Incidence by Year (1973–2005): SEER data versus Model Output.

**Figure 4:**
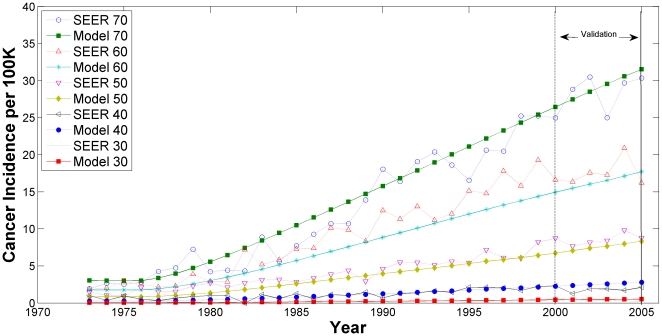
Annual Esophageal Adenocarcinoma Incidence: SEER data versus Model Output by Age Group.

The model fit to the SEER cancer incidence, the third calibration target, is presented in Figures 3 and 4. In Fig. 3, the age-adjusted EAC incidence rates are plotted as a function of calendar year for both the Model and SEER data; in Fig. 4, the EAC incidence rates are stratified by specific age groups. All model outputs between years 2001–2005 are for validation.

Age group 20–29 is not shown in Fig. 4 because of the limited resolution in the y-axis; however, the fit was similar or better compared to the other age groups displayed.

### Validation Results

The adjusted GOF scores (adjusted for number of years in the period) of the superior (top 1%) parameter sets were calculated for the validation years (2001–2005) and compared to the adjusted GOF scores from the calibration years (1973–2000). The adjusted GOF value for the validation set met criterion and even exceeded expectations as it was lower than the GOF value for the calibration set with a value of 1.159 compared to 1.30, respectively, confirming model validation.

### Aspirin Chemoprevention Analysis

**Figure 5:**
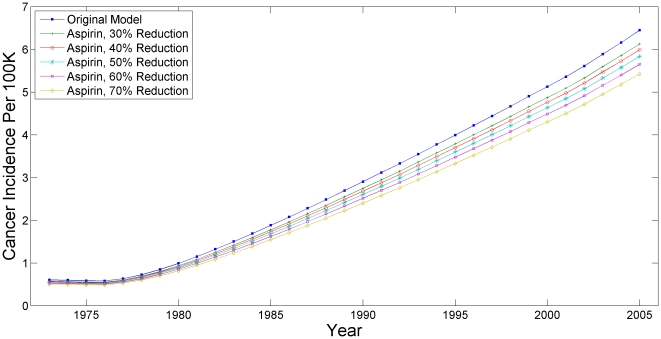
Annual Cancer Incidence with Aspirin Chemoprevention.

The effect of a national aspirin chemoprevention program (all white men age 40 and older) starting in 1960 is presented as a graph in Fig. 5. The top line shows the base case result and represents the scenario of aspirin resulting in a 50% reduction in 5 year cumulative EAC incidence. A sensitivity analysis varied the potential efficacy of aspirin from 30–70% with results graphed alongside the base case 50% reduction for ease of comparison. The number of prevented cancers per year was estimated by using the top 1% of parameter sets and calculating an average value, presented with the range of predicted values.

The overall results of the base case 50% reduction are summarized in Table 3. Notably, the range serves as a qualitative indicator of the level of uncertainty in the model prediction. The number of cancers prevented increases with time, which corresponds to the overall rising incidence of EAC during this period.

**Table 3:** EAC Cases Prevented By Aspirin Over Time.

	1975–1980	1981–1985	1986–1990	1991–1995	1996–2000	2001–2005
Original CAs Over 5 Years	2310	4987	8668	13045	17997	24071
CAs From 1975 Onward	2310	7297	15964	29009	47006	71077
Prevented CAs	235 (215–256)	528 (483–574)	904 (829–984)	1315(1205–1435)	1748(1601–1912)	2290(2097–2505)
% Percent Reduction	10.2 (9.3–11.1)	10.6 (9.7–11.5)	10.4 (9.6–11.4)	10.1 (9.2–11.0)	9.7 (8.9–10.6)	9.5 (8.7–10.4)
Prevented CAs From 1975 Onward	235 (215–256)	763 (699–830)	1667(1528–1814)	2982(2733–3249)	4730(4333–5167)	7020(6431–7665)

The *upper* section of the table displays the expected numbers of EAC cases without aspirin, the 1^st^ row is by 5 year period interval and the 2^nd^ row is a cumulative tally.

The *lower* section displays the prevented numbers of cancers for each 5 year period as absolute numbers (1^st^ row) and percentages (2^nd^ row)) and the 3^rd^ row is a cumulative tally of cancers prevented by aspirin. The values in parentheses represent the range found using the superior (top 1%) parameter sets.

Abbreviat

## Discussion

The incidence of EAC in the western world has been rising at an alarming rate. However, the clinical data regarding the EAC natural history is limited. The lack of clear and reliable clinical evidence significantly stymies the development of effective health policy to manage this pernicious cancer. The creation of a simulation model of the natural history of EAC synthesizes the available clinical data creating a unified, coherent, and comprehensive picture or understanding of this disease. The construction of the model has already identified areas, such as the secular trends of age-specific GERD symptom and BE prevalences, where clinical data that are crucial to our understanding of the natural history of EAC are particularly lacking. Future analyses will further highlight and identify these pivotal areas which should be targeted for future research. Any new data that becomes available can be incorporated into the model, thereby allowing it to remain relevant in the future as an integration of the best available data at any time. Finally, our model provides the foundation for future cost-effectiveness analyses to evaluate targeted screening and various management strategies for EAC, a pressing clinical and policy issue as there is currently no accepted strategy.

We present the methods we employed to develop and calibrate a population-based natural history simulation model of EAC. The model was validated as the model fit to validation data was even better than the fit to the calibration target data, exceeding our predefined criterion. We also present the results of an illustrative and exploratory analysis of aspirin chemoprevention using the model to estimate the number of cases of EAC which could have been prevented in the U.S. if aspirin therapy was universally adopted among white males.

Simulation modeling in health care decision analysis is in its early stages. Currently there are no standards for model development or calibration. However a growing consensus espouses the need for a systematic and reproducible method for model calibration [37]. Our EAC model's development included a systematic approach to calibration and validation using GOF scores for quantitative evaluation. Our modeling approach will allow for relatively easy incorporation of future research data into the model, maintaining an up-to-date coherent picture of our current knowledge of the disease's natural history.

Prior mathematical models of BE have analyzed the cost-effectiveness of screening and surveillance for BE, as well as chemoprevention and other treatments in these patients [7], [38], [39], [40], [41]. However, these prior models were single-cohort patient or disease oriented models as opposed to a population model; additionally, they did not fully incorporate SEER data. As a consequence, these models are not suited to analyze national trends in U.S. incidence or to make future projections, limiting their ability to inform public health research and policy decisions.

Our natural history model only included white men because both BE and EAC are most prevalent in white males [1] and the available natural history data from the literature has predominantly focused on this group. A natural history model that included women and other races would suffer from a dearth of clinical and empiric data to perform calibration and validation, creating a model with a high level of uncertainty, mainly useful for exploratory or hypothesis generating analyses.

Although SEER data provided high quality data regarding EAC incidence rates, the other calibration targets (age-specific GERD symptom and BE prevalences) were based on less reliable data from the literature. Specifically, in the absence of convincing data for or against the stability of GERD symptom and BE prevalences over the time period studied, they were assumed to be constant.

Our analysis of SEER data, not reported here, suggests that the stage distribution of EAC's has not appreciably changed over time, implying that the transition rate between Undetected Cancer to Detected Cancer has not changed as well. In order to reproduce the trend of the rapidly rising incidence over the past few decades, our model assumed an increase in the temporal change in the transition rate from BE to Undetected Cancer. Since the Undetected Cancer state is largely an unobservable state, our assumption of constant BE prevalence needs to be verified by future research studies. Epidemiologic data of most cancer incidences often show a flattening or decrease at older ages [24]. Because the biologic mechanism of this phenomenon is not well understood, we did not incorporate this effect into our model to avoid over-fitting, or the potential to fit the model to random error or noise.

As with all models, ours is a simplification of reality. More complicated model structure could be made to increase the clinical accuracy of our model. As an example, we did not include certain clinically relevant health states in the natural history of EAC such as dysplasia (low grade and high grade), as well as stages of cancer. This was in large part due to our decision to incorporate only the essential features for this analysis and to maintain model simplicity for fast computational speed. Other clinical details of EAC will be added to the model in the future versions.

For the illustrative analysis of a national aspirin program effect on EAC incidence, neither the potential cardiac benefits nor the potential harms such as gastrointestinal bleeding were incorporate and complete patient adherence rates was assumed. The purpose of this simple analysis was to provide a relevant and illustrative example; a more detailed analysis would incorporate these and other realities as well as quality of life factors and cost. Consequently, we caution the use of these results to guide clinical recommendations until more conclusive clinical evidence is available.

One advantage of a simulation model is that as new information becomes available (e.g. additional years of SEER data), the model can be updated with relative ease. Aspirin Esomeprazole Chemoprevention Trial (AspECT) is a large randomized controlled trial studying the effects of aspirin and acid suppression therapy on progression rates from BE to EAC [42]. The trial is currently ongoing and will provide high quality trial data not only regarding aspirin, but also about the natural history of BE (control arm). An interim analysis is planned within the next few years and this valuable data could also be incorporated into our model. Future developments to the model could include: the addition of clinically relevant health states such as dysplasia and cancer stage, the incorporation of risk factors, and quality of life utilities and cost. These additions to the model would permit us to conduct effectiveness and cost-effectiveness analyses to evaluate various screening and management strategies for EAC prevention.

## Supporting Information

Appendix S1 (0.18 MB DOC)
