# Correction: Development, Calibration, and Validation of a U.S. White Male Population-Based Simulation Model of Esophageal Adenocarcinoma

**DOI:** 10.1371/annotation/542e1a80-ebeb-400c-bf24-e39afecf4f1a

**Published:** 2010-10-12

**Authors:** Chin Hur, Tristan J. Hayeck, Jennifer M. Yeh, Ethan M. Richards, Stuart J. Spechler, G. Scott Gazelle, Chung Yin Kong

The fourth author's name was spelled incorrectly. The correct name is: Ethan M. Richards. The correct citation is: Hur C, Hayeck TJ, Yeh JM, Richards EM, Spechler SJ, et al. (2010) Development, Calibration, and Validation of a U.S. White Male Population-Based Simulation Model of Esophageal Adenocarcinoma. PLoS ONE 5(3): e9483. doi:10.1371/journal.pone.0009483.

