# Supplementary material for: Development, Calibration, and Validation of a U.S. White Male Population-Based Simulation Model of Esophageal Adenocarcinoma
Source: PLoS One. 2010 Mar 1;5(3):e9483. doi: 10.1371/journal.pone.0009483 (PMC2830429; doi:10.1371/journal.pone.0009483)
Supplement: Appendix S1 — (0.18 MB DOC) [file pone.0009483.s001.doc]

**APPENDIX**

**Assessment of Model fit with GOF**

For each simulation run and distinct parameter set, we computed a Chi-Squared goodness of fit (GOF) score using the following formula:

$$\chi^{2}=\sum_{i=1}^{k} \frac{\left(O_{i}-E_{i}\right)^{2}}{E_{i}}$$

Where *i* is the index for the observation, *E_i_* corresponds to the expected values and *O_i_* corresponds to the observed or simulation result. The upper limit, *k*, corresponds to the total number of observations, and is the product of the number of years and the number of age groups in our analysis.

**Determining the Sequence for the 3 Phase Calibration**

The calibration targets included three endpoints: GERD symptom prevalence, BE prevalence, and SEER cancer incidence. A GOF score could be calculated for a model’s fit to these three calibration endpoints simultaneously. However, the cancer incidence data (SEER) comprises a great number of patients (representative of the US population), the overall model fit calculation would be overwhelmed by the cancer incidence component. To address the heterogeneity of the target endpoint data, the calibration was divided into a three phase sequential process.

Additionally, to determine if the order target endpoint used in the sequential calibration had an effect on model fit, two different sequences were chosen and compared: the biologic or Forward (GERD Symptoms🡪BE🡪EAC); and the Reverse (EAC🡪BE🡪GERD Symptoms) order.

When we performed the Reverse order calibration, it became evident that although the fits to EAC incidence and GERD symptom prevalence were good, the fit to BE prevalence was poor for all of the parameter sets. In contrast, the Forward calibration was able to approximate all three endpoints, demonstrating reasonable fit at each phase of the calibration. In addition, another benefit of the Forward order is its consistency with the biologic sequence of the disease making it intuitively easier to understand and interpret the calibration procedure and results.

**GERD Symptom Prevalence by Age**

Because of the lack of published data on GERD symptom prevalence stratified by age, we started with an overall prevalence of approximately 20% [1]; we then performed a weighted regression on data from all available studies [2,3,4,5,6,7,8,9,10,11,12,13] that presented the mean age and GERD symptoms prevalence of the study group. The regression resulted in an overall prevalence  of 18.6% (weighted average), which is consistent with the US estimate by Locket et al [1] which reported the GERD symptom prevalence in a range between 17.7% - 21.9% (95% confidence intervals). There are few published data regarding GERD symptom prevalence trends over time in the US population. Some have reported the possibility of a rising trend [2]; while, others have suggested that the data is inconclusive, and that even if a trend exists, it would be small in magnitude [14]. In the absence of data to inform a trend, we assumed that the GERD symptom prevalence was constant over time or lacked a secular trend; this assumption was also coherent with our similar assumption that there was no secular trend in BE prevalence.

APPENDIX Figure 1: GERD Symptom Prevalence by Age

**
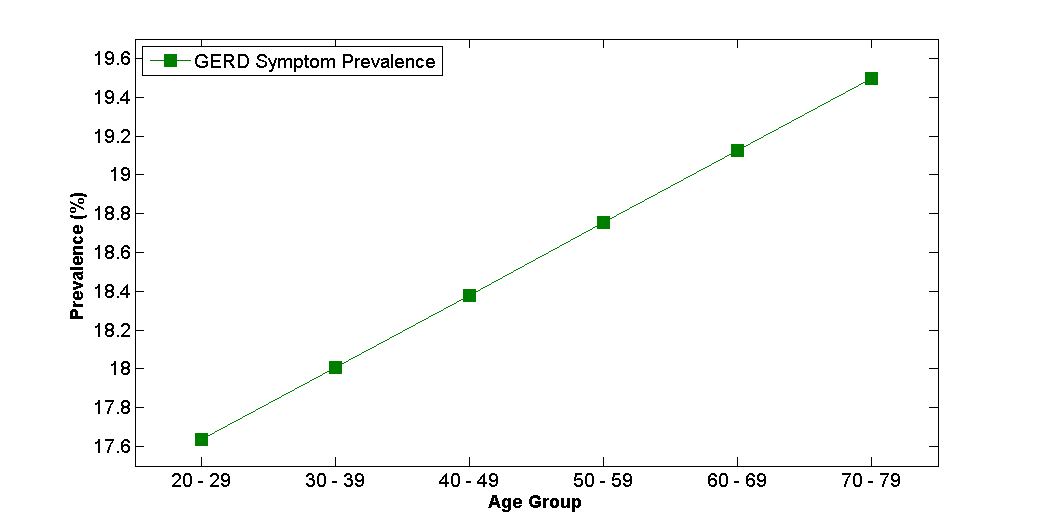
**

**BE Prevalence by Age**

The prevalence of BE in the literature has a wide range (0.8-25%) [8,15,16,17,18,19,20,21,22,23]. The evolving definition of BE with the inclusion of short-segment and ultra-short segment BE has been one of the causes for this ambiguity. The BE prevalences used as the calibration endpoints for our model focused on three specific studies.

The two largest and methodologically most rigorous studies that estimated population prevalences of BE were published by Rex et al [15] and Ronkainen et al [8]. The third study we used was published in 1993 and reports one of the lowest estimates for BE prevalence (0.8%) in the literature, but was the only source of age-stratified data [24]. The Ronkainen study, which was performed in Sweden, had a superior community sampling methodology than the Rex study, where the study cohort consisted of patients who came to an endoscopy center for a colonoscopy. However, the 6.8% prevalence of BE found by the Rex study is more consistent with the higher prevalences in recent US studies [21,22] than the significantly lower 1.6% prevalence reported by Ronkainen’s group. It has been hypothesized that differing diagnostic criteria (e.g. endoscopic biopsy methods) could have contributed to the divergent findings. Consequently, we chose to average the results of these two similar sized studies and estimated that the overall prevalence of BE in the US population was approximately 4.2% in the base case. We then performed a linear regression on the age-specific BE prevalences from the Cameron article and recorded the resulting regression slope. This slope was used to generate our age dependent BE calibration target with an overall prevalence of 4.2% as shown in the figure below.

APPENDIX Figure 2: BE Prevalence by Age


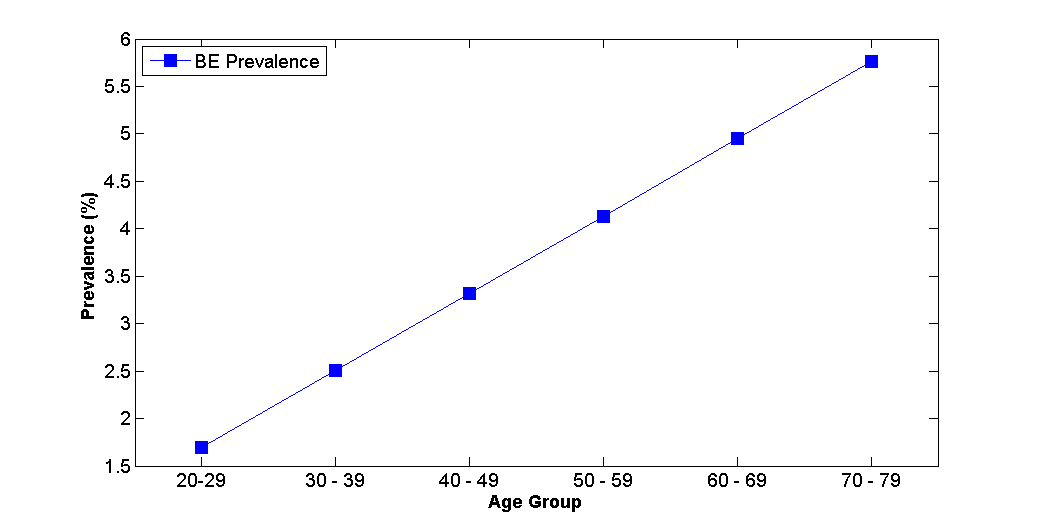


Secular Trend Function

As mentioned in the Methods section, because the prevalence of BE was assumed to be stable over time while EAC incidence exhibits a rapidly rising secular trend, the transition probability from BE to Undetected Cancer required a secular effect for the model output to approximate SEER cancer incidence.

Without knowing the general form of the time trend (e.g. linear, power, polynomial, etc.) sample simulations were run to test the fits of various possible trends. For these simulations all of the input parameters where held constant except for the time factor term. For example, if progression from BE to Undetected cancer was previously represented by the equation: *t = f*(*a*) where *t* is the transition rate from BE to Undetected Cancer and *a* is the age term, then an additional term was added for calendar year: *t = P*(*y*)**f*(*a*) where *P*(*y*) is a pre-factor function and *y* is the year. The goal was to find a function for *P*(*y*) that fit the calibration targets.

We attempted to fit the function *P*(*y*) using linear, power, and polynomial functions; however, the various functional forms produced similar results, with corresponding R^2^ values approximately 0.95. In this setting of equivalent performance, we chose the linear function as it was the simplest. The smallest number of terms in the function made it the most transparent and the most computationally efficient for the calibration process.

**Estimates of Transition Probabilities**

As mentioned in the Methods section, the annual transition probability from BE to Undetected Cancer is difficult to obtain from clinical observations. To allow other researchers to utilize our simulation results, we report estimations of this annual transition probability obtained from our model calibration in Appendix Figures 3A and B. For both graphs, the transition probabilities for each category were calculated based on the top 1% (or 1,000) parameter sets. Appendix Figure 3A shows the distribution of BE to undetected cancer transition probabilities with the calendar year constant or fixed in 1986. We chose to present the plots of three different age groups (20, 50 and 70) and found that the progression rate increased with age. Appendix Figure 3B presents the distribution of BE to undetected cancer transition probabilities with the age constant at 50 for calendar years 1973, 1986, and 2005. The BE to undetected cancer transition probability’s rise correlates to increases in calendar year (or with the latter periods). Additionally, an increase in either age or calendar year also results in greater variability in the range of the distributions.

**Appendix Figures 3, A & B: Barrett’s Esophagus to Undetected Cancer Transition Probability Distributions comparing Age (Fig. 3A) and Year (Fig. 3B)**

| **A)** | **B)** |
| --- | --- |
| **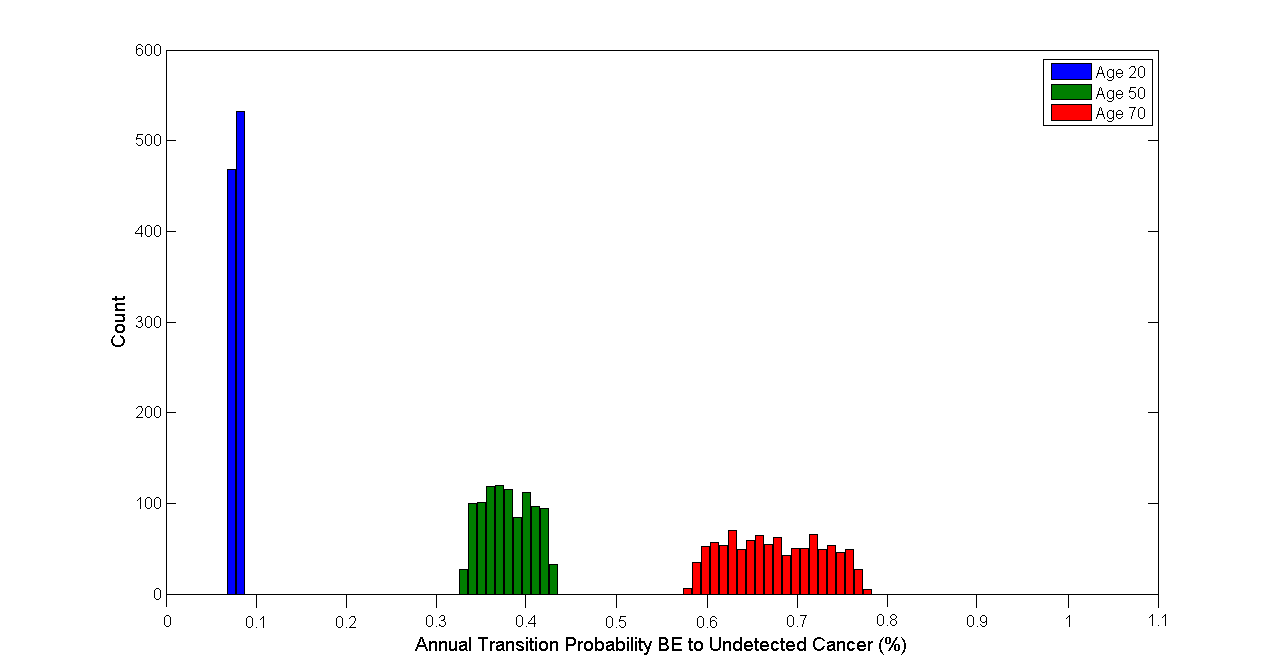** | **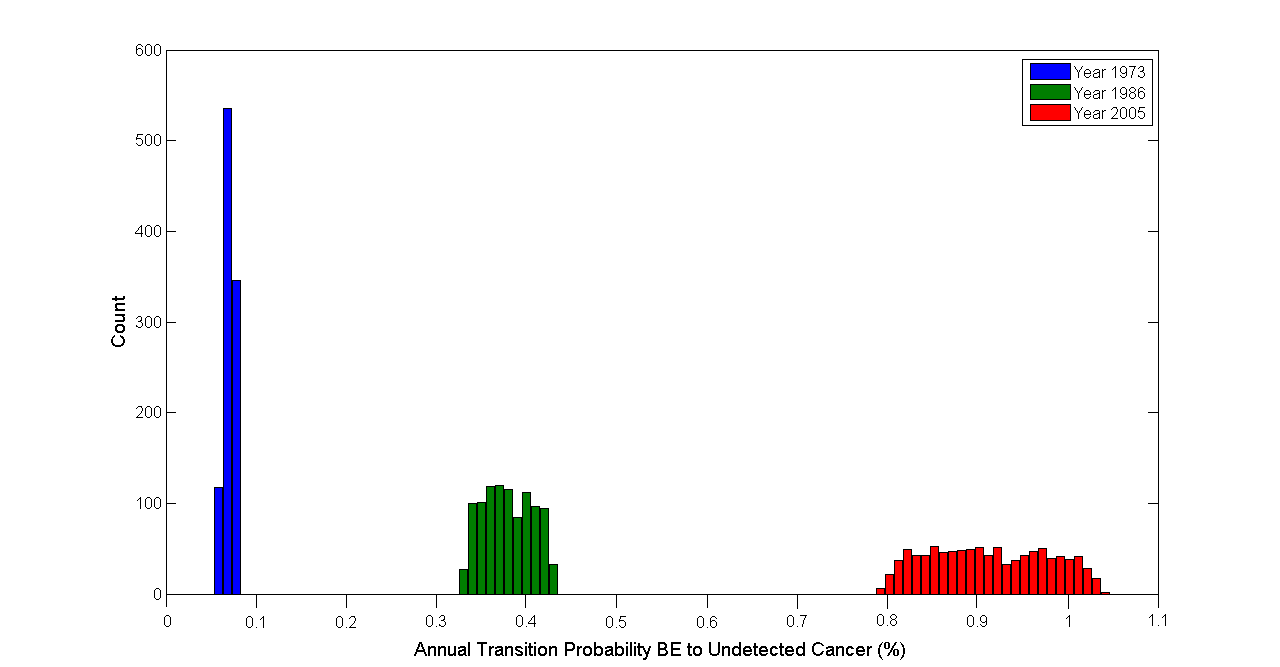** |

**Appendix References:**

1. Locke 3rd GR, Talley NJ, Fett SL, Zinsmeister AR, Melton 3rd LJM (1997) Prevalence and clinical spectrum of gastroesophageal reflux: a population-based study in Olmsted County, Minnesota. Gastroenterology 112: 1148-1156.

**2. El-Serag HB (2007) Time Trends of Gastroesophageal Reflux Disease: A Systematic Review. Clinical Gastroenterology and Hepatology 5: 17-26.**

**3. Valle C, Broglia F, Pistorio A (1999) Prevalence and impact of symptoms suggestive of gastroesophageal reflux disease. Digestive Diseases and Sciences 44: 1848 - 1852.**

**4. Chiocca JC, Olmos JA, Salis GB, Soifer LO, Higa R, et al. (2005) Prevalence, clinical spectrum and atypical symptoms of gastro-oesophageal reflux in Argentina: a nationwide population-based study. Alimentary Pharmacology and Therapeutics 22: 331 - 342.**

**5. El-Serag HB, Petersen NJ, Carter J, Graham DY, Richardson P, et al. (2004) Gastroesophageal Reflux Among Different Racial Groups in the United States. Gastroenterology 126: 1692-1699.**

**6. Isolauri J, Laippala P (1995) Prevalence of Symptoms Suggestive of Gastroesophageal Reflux Disease in an Adult Population. Annals of Internal Medicine 27: 67 - 70.**

**7. Mohammed I, Cherkas LF, Riley SA, Spector TD, Trudgill NJ (2003) Genetic influences in gastro-oesophageal reflux disease: a twin study. Gut 52: 1085 - 1089.**

**8. Ronkainen J, Pertti A, Storskrubb T, Johansson S-E, Linde T, et al. (2005) Prevalence of Barrett's esophagus in the general population: an endoscopic study. Gastroenterology 129: 1825-1831.**

**9. Terry P, Lagergren J, Wolk A, Nyren O (2000) Reflux-Inducing Dietary Factors and Risk of Adenocarcinoma of the Esophagus and Gastric Cardia. Nutrition and Cancer 38: 186 - 191.**

**10. Locke GR, 3rd, Talley NJ, Fett SL, Zinsmeister AR, Melton LJ, 3rd (1997) Prevalence and clinical spectrum of gastroesophageal reflux: a population-based study in Olmsted County, Minnesota. Gastroenterology 112: 1448-1456.**

**11. Collen MJ, Abdulian JD, Chen YK (1995) Gastroesophageal Reflux Disease in the Elderly: More Severe Disease That Requires Aggressive Therapy. American Journal of Gastroenterology 90: 1053 - 1057.**

**12. Talley NJ, Zinsmeister AR, Schleck CD, Melton 3rd LJ (1992) Dyspepsia and Dyspepsia Subgroups: A Population-Based Study. Gastroenterology 102: 1259 - 1268.**

**13. Diaz-Rubio M, Moreno-Elola-Olaso C, Rey E, Locke 3rd GR, Rodriguez-Artalejo F (2004) Symptoms of gastro-oesophageal reflux: prevalence, severity, duration and associated factors in a Spanish population. Alimentary Pharmacology and Therapeutics 19: 95 - 105.**

**14. Shaheen N, Provenzale D (2003) The epidemiology of gastroesophageal reflux disease. Am J Med Sci 326: 264-273.**

**15. Rex DK, Cummings OW, Shaw M, Cumings MD, Wong RK, et al. (2003) Screening for Barrett's esophagus in colonoscopy patients with and without heartburn. Gastroenterology 125: 1670-1677.**

**16. Cameron AJ, Lomboy CT (1992) Barrett's esophagus: age, prevalence, and extent of columnar epithelium. Gastroenterology 103: 1241-1245.**

**17. Clark GWB, Ireland AP, Peters JH, Chandrasoma P, DeMeester TR, et al. (1997) Short-segment Barrett's esophagus: A prevalent complication of gastroesophageal reflux disease with malignant potential. Journal of Gastrointestinal Surgery 1: 113-122.**

**18. O'Connor JB, Falk GW, Richter JE (1999) The incidence of adenocarcinoma and dysplasia in Barrett's esophagus: report on the Cleveland Clinic Barrett's Esophagus Registry. Am J Gastroenterol 94: 2037-2042.**

**19. Corley DA, Levin TR, Habel LA, Weiss NS, Buffler PA (2002) Surveillance and survival in Barrett's adenocarcinomas: a population-based study. Gastroenterology 122: 633-640.**

**20. Corey KE, Schmitz SM, Shaheen NJ (2003) Does a surgical antireflux procedure decrease the incidence of esophageal adenocarcinoma in Barrett's esophagus? A meta-analysis. Am J Gastroenterol 98: 2390-2394.**

**21. Westhoff B, Brotze S, Weston A, McElhinney C, Cherian R, et al. (2005) The frequency of Barrett's esophagus in high-risk patients with chronic GERD. Gastrointest Endosc 61: 226-231.**

**22. Gerson LB, Shetler K, Triadafilopoulos G (2002) Prevalence of Barrett's esophagus in asymptomatic individuals. Gastroenterology 123: 461-467.**

**23. Pera M (2003) Trends in incidence and prevalence of specialized intestinal metaplasia, barrett's esophagus, and adenocarcinoma of the gastroesophageal junction. World Journal of Surgery 27: 999-1008.**

**24. Cameron AJ (1993) Epidemiologic studies and the development of Barrett's esophagus. Endoscopy 25: 635-636.**
